# Epileptiform activity predicts epileptogenesis in cerebral hemorrhage

**DOI:** 10.1002/acn3.51637

**Published:** 2022-08-27

**Authors:** Tseun Han James Kong, Mohammad Abdul Azeem, Ayesha Naeem, Shawn Allen, Jennifer Ahjin Kim, Aaron F. Struck

**Affiliations:** ^1^ Department of Neurology University of Wisconsin‐Madison Madison Wisconsin USA; ^2^ Department of Neurology Yale University New Haven Connecticut USA; ^3^ William S. Middleton Veterans Administration Hospital Madison Wisconsin USA

**Keywords:** epilepsy, cerebral hemorrhage, EEG, neuro‐monitoring

## Abstract

This retrospective case–controlled study was performed to evaluate whether Epileptiform Activity, suspected clinical seizures, and/or 2HELPS2B/S after nontraumatic Intraparenchymal Hemorrhage or Subarachnoid Hemorrhage can predict Epilepsy. Hundred and thirty‐two patients were included—29 (Epilepsy), 103 (Control Group). After matching, the average effect for all three risk factors was significant as follows: (1) Epileptiform Activity (*p* = 0.012, odds ratio 3.14), (2) suspected seizures (*p* = 0.021, odds ratio 3.78), and (3) 2HELPS2B/S score (*p* < 0.001, odds ratio 4.94). This study shows that Epileptiform Activity, suspected seizures, and particularly, the 2HELPS2B/S score in the acute phase are risk factors for the development of epilepsy after nontraumatic intraparenchymal and subarachnoid hemorrhage.

## Introduction

Brain injury is a common cause of epilepsy and results in decreased quality of life and increased mortality.^1^ The latent period between brain injury and epilepsy is a target for antiepileptogenesis therapy. Postinjury epilepsy is an attractive research area as it has animal model analogs, is a capturable population for trials, and has a window for intervention. Despite success in animal models, the clinical trial results have been disappointing.^2^ One element that may improve future trials is enriching the study population with those most likely to develop epilepsy. Developing biomarkers that can risk stratifying postinjury patients has evident clinical utility and can improve clinical trial efficacy.^3^

One potential biomarker is electroencephalogram (EEG) Epileptiform Activity. These EEG abnormalities have long been recognized as a harbinger of epilepsy in animal models^4^ and were recently found to be associated with an increased risk of posttraumatic epilepsy.^5^ Similarly, acute symptomatic seizures are also associated with an increased risk of epilepsy after ischemic stroke.^6^ Here we expand upon these findings in a case–controlled study in 132 patients with either subarachnoid hemorrhage (SAH) or intraparenchymal hemorrhage (IPH) with covariate adjustment with coarsened exact matching to determine if epileptiform abnormalities, suspected acute symptomatic seizures, and a seizure risk score—2HELPS2B/S^7^ can predict the development of epilepsy. Further, we examine the influence of antiseizure medications (ASM) on the risk of developing epilepsy.

## Methods

**Figure 1:**
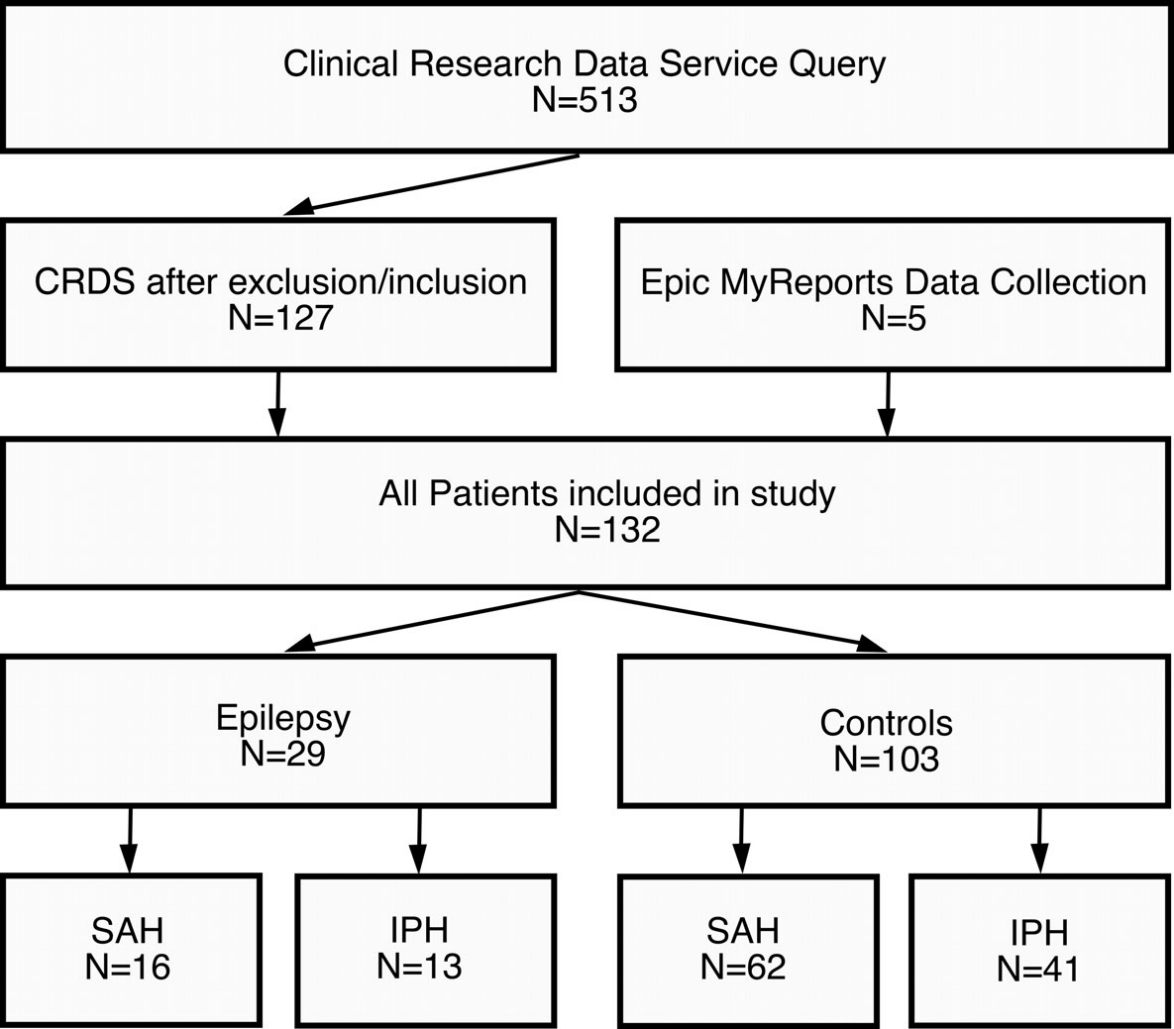
Flowchart showing the numbers of participants identified through the screening process. 513 patients were identified by billing codes, and five patients were identified through a separate process. Inclusion and exclusion criteria were applied, and 132 patients were ultimately included in the study.

This retrospective case–controlled study was approved by the institutional review board (IRB) at the University of Wisconsin—Madison. Waiver for informed consent was granted. Medical records were reviewed for adult patients presenting between 2003 and 2018 with either subarachnoid hemorrhage or intraparenchymal hemorrhage. Most of the patients were identified via our Clinical Research Data Service Query (CDRS) which utilized an algorithmic search of billing codes with the following criteria: (1) patient has an inpatient admission with a final hospital billing diagnosis that includes SAH (ICD codes 430, I60, I69) or intracerebral hemorrhage (ICD codes 431, I61), (2) patient has a billing code for EEG associated with the admission, (3) discharge date within the time frame January 1, 2010 through September 30, 2018, and (4) the patient is 18 years or older on the discharge date. The initial CRDS algorithm yielded 513 patient encounters. Prior to 2010, a separate data collection strategy was employed using the Epic “My Reports” tool using parameters that identified patients who had an EEG and were admitted by the Neurosurgeons at our institution. Both sets of patients were then screened for inclusion and exclusion criteria. Study inclusion criteria included (1) age ≥18, (2) nontraumatic SAH or IPH at presentation, (3) routine or continuous EEG monitoring during initial hospital admission within 14 days of presentation, (4) for controls—a clinic follow‐up 2 years following the presentation, where it is determined that a patient did not develop epilepsy. Study exclusion criteria included (1) known diagnosis of seizures/Epilepsy, (2) presentation with subdural hematomas, (3) cause of hemorrhage is a conversion from ischemic stroke, (4) recent trauma/surgery, known intracranial neoplasm. After combining both sets of patients, 132 patients were included in the study (Fig. 1). Subjects are considered to have developed Epilepsy if they have at least one documented clinical seizure between 1 and 24 months. The remaining patients were identified as controls. Given the above criteria, all the participants in this study had a full data set except for four controls with missing ASM data.

Three risk factors and ASM use were selected a priori and analyzed to assess their influence on epilepsy development. *Epileptiform activity* was defined as the presence of EEG markers of cortical irritability within 2‐week postinjury. These EEG markers include sporadic epileptiform discharges, seizures, brief potentially ictal rhythmic discharges (BRDs), Lateralized Periodic Discharges (LPDs), Generalized Periodic discharges (GPDs) if >2 Hz or plus features, and Lateralized Rhythmic Delta Activity (LRDA). The second risk factor was *suspected acute symptomatic seizure* at presentation. The third risk factor was *2HELPS2B/S*.^7^ *2HELPS2B/S* is a modified form of 2HELPS2B. The difference is that either BRDs or electrographic seizures will net two points as the neurophysiology of BRDs and electrographic seizures are similar.^8^ The purpose of *2HELPS2B/S* was to quantify cortical irritability. ASM variable was defined as being on an ASM either at the time of the first unprovoked epileptic seizure (for subjects) or use of ASM at a 2‐year follow‐up (for controls).

Univariate comparison prior to matching was performed on risk factors and covariates (Table 1). Statistical analysis was performed with permutation testing. Commonly used clinical break points for continuous variables (intraparenchymal hemorrhage volume) or clinical ordinal scales were defined a priori and used to dichotomize variables.

**Table 1:** Unmatched patient characteristics and Matched Risk Factor analysis.

Unmatched patient characteristics—*n* (%)			
	No Epilepsy	Epilepsy	*p* value
Demographics			
Age in years (mean)	58.3	54.7	0.23
Age ≥65 years	34 (33)	8 (27)	0.74
Sex (76 female, 57.6%)	59 (57)	17 (59)	1
IPH and SAH (*N* = 132)	103 (100)	29 (100)	
IPH (*N* = 54)	41 (40)	13 (45)	
SAH (*N* = 78)	62 (60)	16 (55)	
Clinical factors			
IPH versus SAH (% with IPH)	41 (40)	13 (48)	0.55
ICH score >2	8 (20)	3 (23)	0.85
HH >2	19 (31)	8 (50)	0.32
Fisher >2	53 (86)	16 (100)	0.24
Intraventricular hemorrhage	42 (68)	11 (69)	1
Hydrocephalus	41 (66)	14 (88)	0.17
GCS (mean)	11.1	10.4	0.44
GCS <8	31 (30)	11 (38)	0.57
ASM at time of seizure or 2‐year follow‐up	28 (27)	14 (48)	0.058
ASM at time of EEG	98 (95)	28 (96)	1
EEG Findings			
Day of the first EEG (mean)	4.1	3.3	0.29
EEG duration hours (mean)	23.06	47.7	0.007
Electrographic seizure	3 (3)	4 (14)	0.066
BIRD	0	0	1
Sporadic epileptiform discharge	16 (16)	14 (48)	0.001
Lateralized periodic discharges	3 (3)	3 (10)	0.23
Lateralized rhythmic delta activity	4 (4)	4 (14)	0.13
Generalized rhythmic delta activity	9 (9)	4 (14)	0.65
Focal slowing	51 (50)	16 (55)	0.74
Generalized slowing	85 (83)	24 (83)	1
Burst suppression	9 (9)	4 (14)	0.66
2HELPS2B/S Score (mean)	0.4	1.34	<0.001
Time to first epileptic seizure (median, IQR)		318, 214	
Primary risk factors of interest			
2HELPS2B/S>0	30 (29)	21 (72)	<0.001*
Acute symptomatic clinical seizure	13 (13)	10 (35)	0.042*
Any evidence of EEG cortical irritability	22 (21)	16 (55)	0.003*

Matched risk factor analysis	Average effect estimate		
	OR	Credible intervals	*p* value
Marginal treatment effect			
ASM	1.8	0.40–8.56	0.362
Marginal odds			
Epileptiform activity	3.14	1.11–8.91	0.012*
ASM moderation odds			0.016
Epileptiform activity with ASM	2.08	0.25–9.57	
Epileptiform activity without ASM	3.85	0.62–25.95	
Marginal Odds			
Suspected acute symptomatic seizure	3.79	1.00–14.10	0.021*
ASM moderation odds			0.17
Suspected acute symptomatic seizure with ASM	0.92	0.15–2.30	
Suspected acute symptomatic seizure without ASM	3.1	0.00–29.63	
Marginal odds			
2HELPS2B>0	4.94	1.72–16.64	<0.001*
ASM Moderation Odds			<0.001
2HELPS2B>0 with ASM	3.86	0.82–26.00	
2HELPS2B>0 without ASM	4.53	0.63–36.83	

Abbreviations: ASM, antiseizure medications; EEG, Electroencephalogram; IPH, intraparenchymal hemorrhage; OR, odds ratio; SAH, subarachnoid hemorrhage.

* Adjusted for three multiple comparisons.

Using a case–controlled study design, the distribution across selected covariates was adjusted by coarsened exact matching to estimate the marginal odds ratio. Coarsened exact matching overcomes bias introduced with propensity score with greater flexibility than exact matching.^9^, ^10^ The mean difference in covariates and effective sample size was examined after each matching to assess quality, performed with Matchit.^11^ Covariates adjusted via matching were selected a priori based on the likelihood of affecting the risk factor and the development of epilepsy. The number of matched covariates was minimized and dichotomized along standard clinical break points. Highly correlated variables were excluded (e.g., Hunt‐Hess and GCS). Credible intervals were calculated with bootstrapping with replacement to the full cohort size.^12^ There was no forced balancing between diagnoses. A moderation analysis^13^ was performed to assess the potential influence of ASM on postinjury epilepsy.

The classifier ability of 2HELPS2B/S was analyzed with the area under the ROC curve (AUC).^14^ All analysis was performed in R version 4.1.1 (R Foundation Vienna, Austria). The P value threshold for significance was set at <0.05. For the marginal odds ratio of the three risk factors (primary outcome), a family‐wise error rate was adjusted with the Bonferroni method.

## Results

The final analysis included 132 patients (29 developed epilepsy). Clinical covariates were not statistically different in those who developed epilepsy. However, several EEG factors either had a trend or were statistically significant. The three risk factors of interest were significant: *Epileptiform Activity p* = 0.03, *Suspected Acute Symptomatic Clinical Seizure p* = 0.042, and *2HELPS2B/S*>0<0.001. (Table 1).

**Figure 2:**
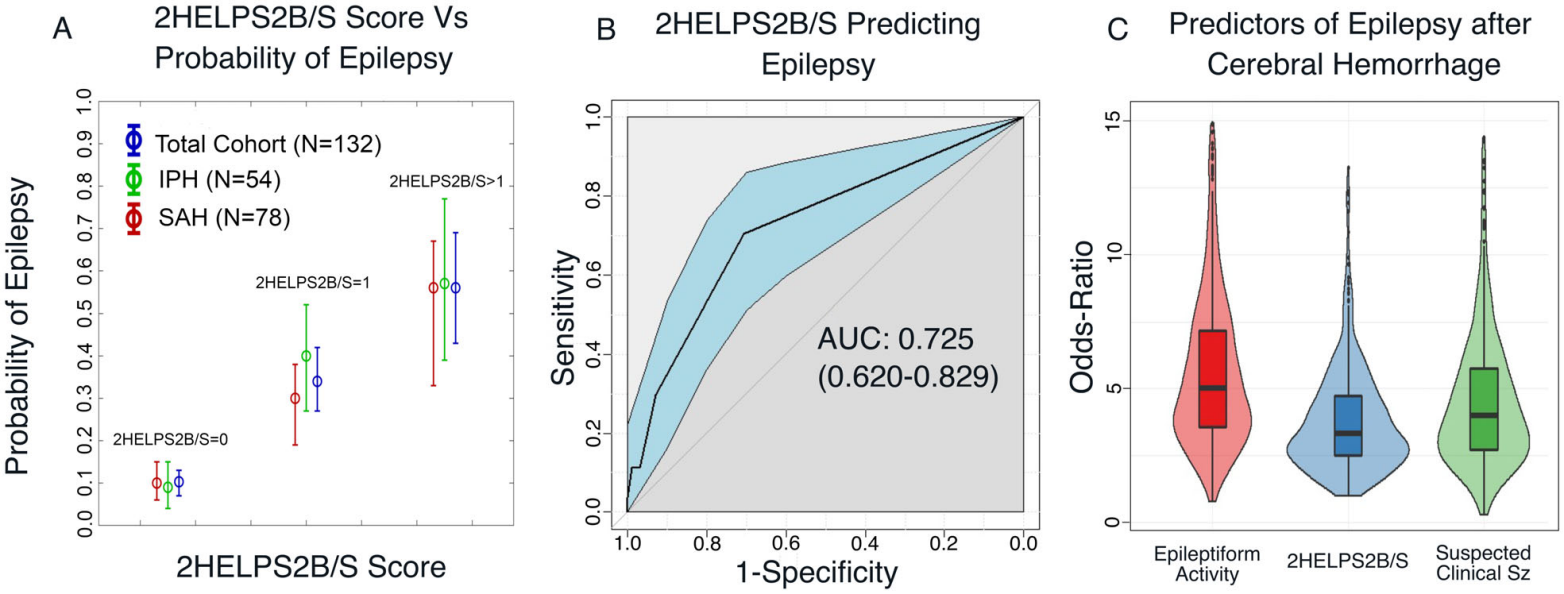
(A) The probability of epilepsy versus 2HELPS2B/S score of 0, 1, or >1 for the whole cohort, IPH, and SAH presented with error bars of ±1 standard deviation. (B) The receiver‐operating characteristic (ROC) with the area under the curve for 2HELPS2B/S to predict the development of epilepsy within 2 years of hemorrhage. (C) A violin plot and a box plot for the three risk factors of epilepsy after nontraumatic subarachnoid hemorrhage or intraparenchymal hemorrhage. The credible intervals and distribution are generated with bootstrap resampling with 500 trials. The risk factors included the following: 2HELPS2B/S (binary with an odds ratio for 2HELPS2B/S>0). Irritable EEG is defined as electrographic evidence of cortical irritability (sporadic epileptiform discharges, lateralized periodic discharges, bilateral independent periodic discharges, lateralized rhythmic delta activity, generalized periodic discharges with a frequency >2 Hz or associated with “plus” features, the presence of brief potentially ictal rhythmic discharges, or electrographic seizures). Suspected‐Clinical‐Sz refers to an event while not on EEG that was reported as a possible or likely clinical seizure at the time of presentation prior to EEG initiation—essentially an acute symptomatic seizure. [Colour figure can be viewed at wileyonlinelibrary.com]

After matching, the marginal treatment effect of prolonged ASM use on the development of epilepsy was not significant (*p* = 0.36). The marginal odds ratio for all three risk factors were significant: *Epileptiform Activity* odds ratio = 3.14, *p* = 0.012; *Suspected Acute Symptomatic Clinical Seizure* odds ratio 3.78, *p* = 0.021; *2HELPS2B/S* odds ratio 4.94, *p* < 0.001 (Fig. 2, Table 1). Matching details are in Supplement A.

The *2HELPS2B/S* score was assessed as a risk factor for the development of postinjury epilepsy in the whole cohort of 132 patients (Fig. 2B‐C) with an AUC of 0.725. Subgroup analysis indicated that there was no difference for 2HELPS2B/S as a predictor of Epilepsy between IPH and SAH groups.

## Discussion

The primary result is that Epileptiform Activity in the acute phase of nontraumatic cerebral hemorrhage is associated with an increased risk of epilepsy. Matching of covariates was performed to minimize the effect of confounders such as hemorrhage size. Additionally, prolonged use of ASM after the acute phase of hemorrhage did not affect epilepsy development. Both Epileptiform Activity and acute symptomatic seizures were significantly associated. The seizure prediction score 2HELPS2B was slightly modified to include electrographic seizures and named 2HELPS2B/S. 2HELPS2B/S combines the other two risk factors and was more powerful than either.

These results are consistent with recent studies. Kim et al. (2018) showed that epileptiform discharges increase the risk of epilepsy after traumatic brain injury.^5^ A large study by Galovic et al. (2018) found acute symptomatic seizures to be the best epilepsy predictor following ischemic stroke.^6^ Similar to the pivotal clinical trial,^2^ ASM did not prevent postbrain injury epilepsy. It remains unclear if the association between Epileptiform Activity in the acute phase of brain injury and the development of epilepsy is causal or related to a similar underlying common cause. Arguing against the hypothesis of causation are the failures of ASM to prevent epilepsy. However, these trials did not identify only the brain‐injured patients with acute cortical irritability nor did they titrate ASM based on individual response to therapy. Future trials looking at antiepileptogenesis therapies should include acute phase EEG monitoring to examine these hypotheses. Even if no causal relationship exists, Epileptiform Activity can still be useful for risk stratification purposes in clinical trials.

Limitations include selection bias, as only patients with 2 years of clinical follow‐up and an EEG were included. It is possible that the requirement for 2 years of follow‐up would disproportionately exclude patients based on insurance, rare, or socioeconomic status. However, we do not have evidence that these differences are related to the development of Epilepsy, and further studies are needed to evaluate these links. An ongoing prospective study (EpiBioSRX^15^) can test these results in a prospective observational study. Matching covariates for case–controlled studies is an imperfect process that does not completely suppress bias; hence, the need for replication. ASM use was also considered a single dichotomized variable due to limits in study size, so an ASM effect based on a dose or a drug mechanism remains possible.

In summary, Epileptiform Activity, suspected acute symptomatic seizures, and particularly the 2HELPS2B/S are promising predictors of postbrain injury epilepsy and should be validated in large prospective observational studies.

## Funding Information

This work was supported by the National Institute of Health (grant no. R01NS111022).

## Authors’ Contribution

Tseun Han James Kong is the first author of this paper and was involved in all aspects of this project except for the statistical analysis itself. Mohammad Abdul Azeem, Ayesha Naeem, and Shawn Allen were directly involved in the acquisition of data as well as the review and editing of this manuscript. Jennifer Ahjin Kim and Aaron F Struck played a vital role in the conception/design of the study and in assisting in drafting this manuscript. Dr Struck was also responsible for performing the statistical analysis.

## Conflict of Interest

No relevant conflict of interest is present for the authors. This study is not a clinical trial and does not use material reproduced from other sources.

## Supporting information


**Supplement A.** Matching Process ‐ Summary of balance for all data with coarsened exact matching.

## Data Availability

Data are available on request due to ethical considerations.
