# Supplementary material for: Epileptiform activity predicts epileptogenesis in cerebral hemorrhage
Source: Ann Clin Transl Neurol. 2022 Aug 27;9(9):1475–80. doi: 10.1002/acn3.51637 (PMC9463945; doi:10.1002/acn3.51637)
Supplement: Supplementary file 1 — Supplement A. Matching Process ‐ Summary of balance for all data with coarsened exact matching. [file ACN3-9-1475-s001.docx]

**Supplement A:** Matching Process

**Matching for Effect of Anti-Seizure Medication to Effect Development of Epilepsy**

Matching was performed on the following covariates: Intra-parenchymal hemorrhage versus subarachnoid hemorrhage (IPHvSAH), Glasgow Coma Scale <8 (gcsBIN), modified Fisher Scale >1 (fisherBIN), Intra-parenchymal hemorrhage volume > 30 mL (ichVOL>30), presence of Epileptiform Activity on EEG within 2 weeks of hemorrhage (eegIrBIN), and a suspected acute symptomatic seizure within 14 days of hemorrhage (onsetSz). The tables below compare the means of the covariates between those treated with ASM, with Controls who were not treated with ASM at 2 year follow-up. Mean Diff is the proportional difference in means. ESS is the effective sample size.

Before Matching

Summary of Balance for All Data:

Treated with ASM Control Mean Diff (fractional)

IPHvSAH (mean) 0.4762 0.3452 0.2622

gcsBIN (mean) 0.4524 0.2619 0.3827

fisherBIN (mean) 0.4762 0.5833 -0.2145

ichVOL>30 (mean) 0.1667 0.1071 0.1597

eegIrBIN (mean) 0.3810 0.2262 0.3187

onsetSz (mean) 0.3571 0.0952 0.5466

After Matching

Summary of Balance for Matched Data:

Treated with ASM Control Mean Diff (fractional)

IPHvSAH (mean) 0.5143 0.5143 0

gcsBIN (mean) 0.4000 0.4000 0

fisherBIN (mean) 0.4571 0.4571 0

ichVOL>30 (mean) 0.2000 0.2000 0

eegIrBIN (mean) 0.3714 0.3714 0

onsetSz (mean) 0.2286 0.2286 0

Sample Sizes:

Control Treated with ASM

All 84 42

Matched (ESS) 37.74 35

Matched 77 35

Unmatched 7 7

Discarded 0 0

**Matching for Epileptiform Activity**

Matching was performed on the following covariates: Intra-parenchymal hemorrhage versus subarachnoid hemorrhage (IPHvSAH), Glasgow Coma Scale <8 (gcsBIN), modified Fisher Scale >1 (fisherBIN), Intra-parenchymal hemorrhage volume > 30 mL (ichVOL>30). The tables below compare the means of the covariates between patients with Epileptiform activity, with Controls without Epileptiform activity. Mean Diff is the proportional difference in means. ESS is the effective sample size.

Before Matching

Summary of Balance for All Data:

Epileptiform activity Control Mean Diff (fractional)

IPHvSAH (mean) 0.4286 0.3736 0.1110

gcsBIN (mean) 0.4857 0.2637 0.4441

fisherBIN (mean) 0.5143 0.5604 -0.0923

ichVOL>30 (mean) 0.1714 0.1099 0.1633

After Matching

Summary of Balance for Matched Data:

Epileptiform activity Control Mean Diff (fractional)

IPHvSAH (mean) 0.4118 0.4118 0

gcsBIN (mean) 0.4706 0.4706 0

fisherBIN (mean) 0.5000 0.5000 0

ichVOL>30 (mean) 0.1765 0.1765 0

Sample Sizes:

Control group Epileptiform activity

All 91 35

Matched (ESS) 69.69 34

Matched 91 34

Unmatched 0 1

Discarded 0 0

**Matching for Suspected Acute Symptomatic Clinical Seizures**

Matching was performed on the following covariates: Intra-parenchymal hemorrhage versus subarachnoid hemorrhage (IPHvSAH), Glasgow Coma Scale <8 (gcsBIN), modified Fisher Scale >1 (fisherBIN), Intra-parenchymal hemorrhage volume > 30 mL (ichVOL>30. The tables below compare the means of the covariates between patients who had Suspected Acute Symptomatic Clinical Seizures (SASCS), with the Controls who did not. Mean Diff is the proportional difference in means. ESS is the effective sample size.

Before Matching

Summary of Balance for All Data:

SASCS Control Mean Diff (fractional)

IPHvSAH (mean) 0.4348 0.3786 0.1133

gcsBIN (mean) 0.5217 0.2816 0.4808

fisherBIN (mean) 0.5652 0.5437 0.0434

ichVOL>30 (mean) 0.0435 0.1456 -0.5009

After Matching

Summary of Balance for Matched Data:

SASCS Control Mean Diff (fractional)

IPHvSAH (mean) 0.4091 0.4091 0

gcsBIN (mean) 0.5000 0.5000 0

fisherBIN (mean) 0.5455 0.5455 0

ichVOL>30 (mean) 0.0455 0.0455 0

Sample Sizes:

Control SASCS

All 103 23

Matched (ESS) 64.19 22

Matched 90 22

Unmatched 13 1

Discarded 0 0

**Matching for 2HELPS2B/S**

Matching was performed on the following covariates: Intra-parenchymal hemorrhage versus subarachnoid hemorrhage (IPHvSAH), Glasgow Coma Scale <8 (gcsBIN), modified Fisher Scale >1 (fisherBIN), Intra-parenchymal hemorrhage volume > 30 mL (ichVOL>30). The tables below compare the means of the covariates between patients who had 2HELPS2B/S>0 and Controls are those who 2HELPS2B/S=0. Mean Diff is the proportional difference in means. ESS is the effective sample size.

Before Matching

Summary of Balance for All Data:

2HELPS2B/S>0 2HELPS2B/S=0 Mean Diff (fractional)

IPHvSAH (mean) 0.4167 0.3718 0.0910

gcsBIN (mean) 0.4375 0.2564 0.3650

fisherBIN (mean) 0.5417 0.5513 -0.0193

ichVOL>30 (mean) 0.1458 0.1154 0.0863

After Matching

Summary of Balance for Matched Data:

2HELPS2B/S>0 2HELPS2B/S=0 Mean Diff (fractional)

IPHvSAH (mean) 0.4043 0.4043 0

gcsBIN (mean) 0.4255 0.4255 0

fisherBIN (mean) 0.5319 0.5319 0

ichVOL>30 (mean) 0.1489 0.1489 0

Sample Sizes:

2HELPS2B/S=0 2HELPS2B/S>0

All 78 48

Matched (ESS) 67.23 47

Matched 78 47

Unmatched 0 1

Discarded 0 0
